# Fishy Curiosities

**Published:** 1882-07

**Authors:** 


### FISHY CURIOSITIES.

Another remarkable property of some of these creatures is the possession of a stomach so capable of distention that it can hold a prey of twice or thrice the bulk of its destroyer. Dr. Günther gives figures of two or three fish with distended stomach ; and Mr. Johnson, his associate in this investigation, writes of a specimen which he procured at Madeira :

"The man from whom I obtained it stated that he had a fish with two heads, two mouths, four eyes, and a tail growing out of the middle of the back, which had astonished the whole market ; and the fishermen, one and all, declared they had never met with anything like it before. At first sight it really did appear to be the monster described ; but a short examination brought to light the fact that one fish had been swallowed by another, and that the features of the former were seen through the extensible skin of the latter. On extracting the fish that had been swallowed, it proved . . . to have a diameter several times exceeding that of its enemy, whose stomach it had distended to an unnatural and painful degree." The action performed by the fish in these cases is not, however, a real swallowing, but more like the similar process executed by serpents.

The interest of Dr. Günther's book does not end with the account of deep-sea fishes, but the chapters devoted to that subject and to classification illustrate the most striking discoveries that have been recently made in ichthyology. Among the curiosities of fish-life that please and amuse as well as instruct, is the story of the fighting-fish of Siam, which on seeing another of its species, or even its own image in a mirror, becomes suddenly excited, and of which, though it is dull in hue at other times, "the raised fins and the whole body shine with metallic colors of dazzling beauty, while the projected gill-membrane, waving like a black frill round the throat, adds something of grotesqueness to the general appearance." The Siamese are infatuated with the combats of these fishes, staking on the results considerable sums, and sometimes their persons and families, while the license to exhibit fish-fights is farmed, and brings in no small revenue to the royal treasury.—*From "Dr. Günther on the Study of Fishes," in Popular Science Monthly for July.*

